# Chronic-disease patients and their use of out-of-hours primary health care: a cross-sectional study

**DOI:** 10.1186/1471-2296-15-114

**Published:** 2014-06-09

**Authors:** Lone Flarup, Grete Moth, Morten Bondo Christensen, Mogens Vestergaard, Frede Olesen, Peter Vedsted

**Affiliations:** 1 Research Unit for General Practice, Department of Public Health, Aarhus University, Bartholins Allé 2, Aarhus DK-8000, C, Denmark; 2 Danish Research Centre for Cancer Diagnosis in Primary Care (CaP), Department of Public Health, Aarhus University, Aarhus, Denmark; 3 Section for General Medical Practice, Department of Public Health, Aarhus University, Aarhus, Denmark

**Keywords:** Out-of-hours services, OOH, Chronic disease, General practice, Primary health care, Reasons for encounter

## Abstract

**Background:**

The general practitioner (GP) plays an important role for chronic disease care. Continuous and close contact with daytime general practice is intended to prevent medical problems arising outside office hours due to already diagnosed chronic disease. However, previous studies indicate that patients with chronic diseases are frequent users of out-of-hours primary care services (OOH), but knowledge is limited on reasons for encounter (RFE), severity of symptoms, and OOH patient handling. We aimed to describe contacts to the OOH services from patients with chronic heart disease, lung disease, severe psychiatric disorders, diabetes, and cancer in terms of RFE, OOH GP diagnosis, assessed severity of symptoms, and actions taken by the GP.

**Methods:**

Eligible patients (aged 18 years and older) were randomly sampled from a one-year cross-sectional study comprising 15,229 contacts to the OOH services in the Central Denmark Region. A cohort of patients with one or more of the five selected chronic diseases were identified by linking data on the Danish civil registration number (CPR) through specific nationwide Danish health registers.

**Results:**

Out of 13,930 identified unique patients, 4,912 had at least one of the five chronic diseases. In total, 25.9% of all calls to the OOH services came from this chronic disease patient group due to an acute exacerbation; 32.6% of these calls came from patients with psychiatric diagnoses. Patients with chronic disease were more likely to receive a face-to-face contact than the remaining group of patients, except for calls from patients with a psychiatric disorder who were more often completed through a telephone consultation. Patients with heart disease calling due to a new health problem formed the largest proportion of all OOH referrals to hospital (13.3%) compared to calls from the other groups with chronic disease (3.4-6.7%).

**Conclusions:**

A third of the patients randomly sampled by their OOH call had one or more of the five selected chronic diseases (i.e. chronic lung disease, heart disease, diabetes, psychiatric disease, or cancer). Patients with chronic disease were more often managed by OOH GPs than other patients.

## Background

Attention has increasingly focused on early detection, efficient treatment, and secondary prevention through proactive follow-up strategies for patients with chronic diseases [1-6]. According to the Danish Health and Medicines Authority, one third of the Danish population lives with at least one chronic disease. Furthermore, treatment and care expenses for these patients account for approximately 80% of the total Danish health-care resources [1,7]. General practitioners (GPs) hold a central role as first-line providers of health care and gatekeepers to specialist care in the Danish tax financed health-care system. GPs offer free specialised family medicine at all hours: during office hours for their listed patients and after hours for patients in acute need of medical care (from 4 pm to 8 am on weekdays and on national holidays) in regionally based out-of-hours (OOH) rota systems in fee-for-service remuneration organizations (OOH) [8,9].

Medical treatment and care of chronic diseases is preferably managed during daytime in general practice. Nevertheless, these patients also consult the OOH [1,2,10,11]. This may be due to the chronic disease, but could also relate to new episodes with other health-care problems. However, we do not know of any previous studies focusing on the extent of use. A recent study showed that half of all patients seen in daytime general practice had chronic diseases; half of their contacts were directly related to the chronic disease [12]. We do not have similar figures for the OOH services, although patients with chronic diseases may form a substantial proportion of frequent attenders at the OOH services [11,13]. More knowledge in this area is a prerequisite for effective planning and quality improvement of the OOH care for patients with chronic diseases. This knowledge is also needed to qualify future incentives, which may transfer some of the OOH contacts into daytime care.

In this study, we therefore aimed to describe the reasons for encounter (RFE) and the diagnoses resulting from contacts to the OOH for patients with five selected highly prevalent chronic diseases (chronic lung disease, chronic heart disease, severe psychiatric disease, diabetes, and cancer) in which disease progression depends on close daytime follow-up by the family GP. Furthermore, we aimed to examine whether the OOH contacts were related to new health problems or to exacerbations of already diagnosed chronic disease. Finally, we also intended to find out whether contacts ended in a telephone consultation, a face-to-face encounter or admission to hospital or emergency department (ED).

## Methods

### Design and setting

The Danish OOH services in primary health care are organised in five regional services covering between 0.6 and 1.8 million residents [14]. In each of the five regions, all patients must first make an initial triage telephone call to the OOH, and all calls are received by a central unit for each region. Except for the Capital Region of Denmark, the telephone triage (in the four other regions) is performed by specially trained GPs. Nurses do, generally, not participate in the OOH care.

The OOH forms part of a fully computerized electronic patient administration system (PAS) in general practice. Nevertheless, the OOH computer system does not have access to day-time patient records unless specific information is sent to the OOH computer. Therefore, the OOH GPs do not have any knowledge of the individual patient’s health background, diagnoses, diagnostic tests, or medications. After each contact to the OOH services, an electronic copy of the patient’s OOH record is sent to the patient’s family GP [15].

The present study is based on information on randomly sampled patient contacts to the OOH in the Central Denmark Region, the largest geographical area of the five Danish regions with approximately 1.3 million residents [14]. These data were originally collected for a Danish cross-sectional survey on disease patterns in the OOH primary health care in the Central Denmark Region during a 12-month period in 2010-2011; the*’Kontakt- og sygdomsmønster i lægevagten LV-KOS2011’* (LV-KOS) [16]. The GPs were electronically invited to participate in the LV-KOS survey when logging on to a duty session in the OOH computer system. Only one GP was permitted to participate per 8-hour shift per contact type, and this implies that a total of 383 GPs (55% of all GPs working at the OOH services during the study period) had the chance to participate in the survey by answering short pop-up questionnaires integrated into the existing electronic OOH patient administration records.

The questionnaires focused on the following themes: whether the RFE was a new event or an exacerbation, duration and severity of symptom(s), possible diagnosis (in text), and estimated relevance of the contact. A total of 21,457 highly representative contacts to the OOH (3.3% of all contacts to the OOH during the 12-month study period) were randomly sampled, and these represented telephone consultations, telephone visitations, face-to-face encounters, and home visits [16].

### Data

The RFEs and diagnoses were obtained from the completed questionnaires, and resulting response data were subsequently coded according to the International Classification of Primary Care, Second Edition (ICPC-2) [17], by specially trained research assistants and validated by one of the authors (LF). Furthermore, data were categorized into either exacerbation of an already diagnosed chronic disease or new health problem. Data on termination of telephone contacts were divided into the following categories: telephone advice, referral to face-to-face encounters or home visit, hospital admission or referral to emergency department (ED), and referral to other authorities (e.g. primary health care, nursing home, or police).

### Data management

The study population comprised only patients aged 18 years or older. We excluded patients without the unique Danish civil registration (CPR) number, which is assigned to all Danish citizens ci. We identified a cohort of patients with chronic lung disease, heart disease, severe psychiatric disease, diabetes, and cancer through correlation of a register-based algorithm for lung disease and diagnostic codes for the other diseases recorded in Danish health registers (Table 1). Collected information included registry data on diagnoses, use of health care services, and prescribed medication for the period from 1 January 2005 to 31 May 2011. The Danish Civil registration number was used to link LV-KOS cohort data with registry data.

**Table 1:** Sources and codes for identification of the chronically ill patients

	**National registries in Denmark**	**Variables**
**Heart diseases**	Danish National Registry of Patients	ICD-10 codes: I20, I21
**Lung diseases**	Danish National Registry of Patients	ICD-10 codes: J40-47, J96
	National Health Insurance Service Registry	Service codes: 7113, 7121 (spirometries)
	Danish National Prescription Registry	ATC codes: R03AC, R03AK, R03BA, R03BB, R03CC, R03DA, R03DC, V03AN01 (oxygen)
**Diabetes**	National Diabetes Registry	Civil registration number (CPR) and corresponding registry data
**Psychiatric diseases**	Danish Psychiatric Central Registry	ICD-10 codes: F20, F25, F30, F31
**Cancer**	Danish Cancer Registry	ICD-10 codes: C00-C97 (excl. C44)

Patients with heart disease, i.e. unstable angina (ICD-10 code I20) and post-myocardial infarction (ICD-10 code I21), were identified in the Danish National Patient Registry [18]. Patients with diabetes were identified if registered in the National Diabetes Registry [19].

Patients with severe psychiatric disease, i.e. schizophrenia (ICD-10 code F20), schizoaffective disorders (ICD-10 code F25), and bipolar affective disorder (ICD-10 codes F30 and F31), were identified in the Danish Psychiatric Central Registry [20]. Patients with cancer were identified in the Danish Cancer Registry (ICD-10 codes C00-C97, excluding non-melanoma cancers (C44)) [21,22]. As many patients with chronic lung disease are not registered in the Danish National Patient Registry if they have not been hospitalised due to their lung disease, this group of patients was identified by an algorithm [23] comprising ICD-10 diagnoses J40-J47 or J96, *or* redemption of at least two prescriptions defined by the ATC codes R03AC, R03AK, R03BA, R03BB, R03CC, and R03DA, R03DC (all including sub-codes) within the past 12 months, *or* oxygen treatment with the ATC code V03AN01, *or* having at least two spirometries with service codes 7113, 7121 within the past 12 months. A subgroup of patients with two or more of the five chronic diseases (2 + diagnosed) was also identified. Patients with more than one of the five chronic diseases were included in the numerator for each specific disease.

### Statistical analyses

Descriptive analyses were performed for each of the five disease group (irrespective of individuals with multiple diagnoses) and for the 2 + diagnosed patients. The population was divided in accordance with the OOH GP’s assessment as to whether the contact was caused by a chronic disease or by a new health problem. Data are presented as frequencies with corresponding percentages. Data were analysed using STATA 11.0 (StataCorp LP, College Station, TX, USA).

### Ethical approvals

The project was approved by the Danish Data Protection Agency (J.no. 2011-41-6365). According to Danish law, approval by the National Committee on Health Research Ethics was not required as no biomedical intervention was performed in this study.

## Results

From the LV-KOS sample of 21,457 contacts to the OOH, we included 15,229 contacts (71%) from patients aged 18 years or older, and this study group represented a total of 13,930 unique patients (70.2%). We thereby excluded 5,433 patients under 18 years of age and 489 patients without a Danish civil registration number. The study population then consisted of 13,930 unique adults of whom 3,624 (26.0%) had one of the five selected chronic diseases and 1,288 (9.2%) had two or more (Table 2). The majority of the patients (94.8%) had only one telephone call to the OOH during the study period. The most frequent single diagnoses for patients with only one of the chronic diseases were lung disease (31.7%), diabetes (26.6%), and cancer (29.0%). The most common combinations of diagnoses among the total study population were lung disease and diabetes (1.9%), lung disease and cancer (1.7%), heart disease and diabetes (0.7%), heart disease and lung disease (0.6%), and diabetes and cancer (0.5%).

**Table 2:** **The proportion of patients with chronic diseases distributed over contact types**^**1**^

	**Telephone consultation**		**Telephone referral**		**Clinical consultation**		**Home visit**		**All**	
**The unique patients are diagnosed to:**	**n**	**(%)**	**n**	**(%)**	**N**	**(%)**	**N**	**(%)**	**n**	**(%)**
0: None of the five diagnoses	2,076	(70.3)	1,322	(65.0)	3,049	(79,1)	2,571	(50.5)	9,018	(64.9)
1: Only one of the five diagnoses	694	(23.5)	549	(27.0)	674	(17.5)	674	(33.5)	3,624	(25.9)
2: Two or more of the five diagnoses	183	(6.2)	162	(8.0)	130	(3.4)	813	(16.0)	1,288	(9.2)
Total	2,953	(100.0)	1,975	(100.0)	3,853	(100.0)	5,152	(100.0)	13,930	(100.0)

^1^The number of identified patients for each contact type is calculated without consideration of patients with more than one of the chronic diseases; consequently, the total will sum up to more than 13,930 unique patients with chronic diseases.

More women (54.5%) than men (45.5%) contacted the OOH services (Table 3). This distribution was also observed for each individual chronic disease, except for heart disease (42.0%) and diabetes (46.4%) where less women had taken contact to the OOH services. Contrary to other chronic diseases, the proportion of patients with psychiatric diseases decreased with age; hence, most patients with psychiatric diseases were aged 18-40 years (50.8%). For patients with heart disease, diabetes, or cancer, the largest proportion of patients who contacted the OOH was patients aged 75 years or older as these groups represented 47.6%, 30.9%, and 35.3%, respectively.

**Table 3:** Age and gender distribution of patients with chronic diseases and of the total population

	**Patients with one chronic disease**										**Patients with two or more chronic diseases**		**All patients with chronic diseases**		**Total population**	
	**Heart disease**		**Lung disease**		**Diabetes**		**Psychiatric disease**		**Cancer**							
Gender (n (%))																
Male	134	(58.0)	500	(43.9)	512	(53.6)	125	(49.2)	374	(36.8)	582	(45.2)	2,227	(45.3)	6,334	(45.5)
Female	97	(42.0)	639	(56.1)	443	(46.4)	129	(50.8)	671	(64.2)	706	(54.8)	2,685	(54.7)	7,596	(54.5)
All	231	(100.0)	1,139	(100.0)	955	(100.0)	254	(100.0)	1,045	(100.0)	1,288	(100.0)	4,912	(100.0)	13,930	(100.0)
Age groups in years (n (%))																
18-40	7	(3.0)	273	(24.0)	125	(13.1)	129	(50.7)	183	(17.5)	59	(4.6)	776	(15.8)	5,331	(38.3)
41-60	51	(22.1)	315	(27.7)	264	(27.6)	88	(34.7)	217	(20.8)	258	(20.0)	1,193	(24.3)	3,503	(25.2)
61-75	63	(27.3)	261	(22.8)	271	(28.4)	31	(12.2)	276	(26.4)	444	(34.5)	1,346	(27.4)	2,387	(17.1)
+75	110	(47.6)	290	(25.5)	295	(30.9)	6	(2.4)	369	(35.3)	527	(40.9)	1,597	(32.5)	2,709	(19.5)
All	231	(100.0)	1,139	(100.0)	955	(100.0)	254	(100.0)	1,045	(100.0)	1,288	(100.0)	4,912	(100.0)	13,930	(100.0)

The highest proportion of calls completed by a telephone consultation was found for patients with psychiatric diseases (57.1% and 58.3%) compared to the other patient groups, implying that patients with psychiatric diseases were less often referred to a face-to-face encounter (Table 4).

**Table 4:** **Telephone calls**^**1 **^**from patients with chronic disease**^**2 **^**and from other patients according to reason for encounter**^**3**^

	**Heart disease (N = 156)**		**Lung disease (N = 627)**		**Diabetes (N = 607)**		**Psychiatric disease (N = 219)**		**Cancer (N = 597)**		**2+ diagnoses (N = 403)**		**Remaining population (N = 3,557)**	
**New health problem (n (%))**	**n = 120 (76.9)**		**n = 455 (72.6)**		**n = 451 (74.3)**		**n = 147 (67.1)**		**n = 458 (76.7)**		**n = 293 (72.7)**		**n = 3,143 (88.4)**	
Telephone cons.	44	(36.7)	207	(45.5)	199	(44.0)	84	(57.1)	209	(45.6)	128	(43.7)	1,663	(52.9)
Referral to OOH face-to-face encounter	53	(44.2)	199	(43.7)	196	(43.5)	49	(33.3)	208	(45.4)	129	(44.0)	1,223	(38.9)
Referral to hospital/ED	16	(13.3)	23	(5.1)	30	(6.7)	5	(3.4)	21	(4.6)	20	(6.8)	92	(2.9)
Other ^4^	7	(5.8)	26	(5.7)	26	(5.8)	9	(6.1)	20	(4.4)	16	(5.5)	165	(5.3)
Total	120	(100.0)	455	(100.0)	451	(100.0)	147	(100.0)	458	(100.0)	293	(100.0)	3,143	(100.0)
Exacerbation of a chronic disease (n (%))	n = 36 (22.5)		n = 172 (27.4)		n = 138 (25.7)		n = 72 (32.9)		n = 139 (23.3)		n = 110 (27.3)		n = 414 (11.6)	
Telephone cons.	15	(41.7)	59	(34.3)	71	(45.5)	42	(58.3)	55	(39.6)	44	(40.0)	222	(53.6)
Referral to OOH face-to-face encounter	18	(49.9)	104	(60.4)	76	(47.7)	24	(33.3)	74	(53.2)	59	(53.6)	169	(40.8)
Referral to hospital/ED	1	(2.8)	7	(4.1)	3	(1.9)	3	(4.2)	6	(4.3)	2	(1.8)	10	(2.4)
Other^5^	2	(5.6)	2	(1.2)	6	(3.9)	3	(4.2)	4	(2.9)	5	(4.6)	13	(3.1)
Total	36	100.0	172	(100.0)	156	(100.0	72	(100.0)	139	(100.0	110	(100.0)	414	(100.0)

^1^Telephone consultations and telephone referrals. ^2^Sum of contacts for diagnostic groups exceeds actual number of contacts for due to multi-morbidity. ^3^A total number of 4,986 unique patients. ^4^Enquiries, etc. ^5^Contact to primary care, nursing homes, police, etc.

No marked proportional differences were found between the five diagnostic groups and the remaining population for calls caused by a new health problem handled exclusively as a telephone consultation. The proportion of patients who were referred to hospital admission within each disease group (except for psychiatric disease) was either the same or higher for patients who had a contact to the OOH due to a new health problem than for those with an exacerbation; for patients with psychiatric disease, the opposite was observed. The largest proportion of contacts referred to hospital admission came from patients with heart disease contacting the OOH due to a new health problem (13.3%). This proportion was noticeably higher in the group of 2 + diagnosed patients (6.8%) than proportions found for the other four diseases (3.4-6.7%) and in the remaining population (2.9%) (Table 4).

Table 5 shows the five most frequent RFEs for each of the five chronic diseases. Telephone consultations for medical prescriptions were one of the three most common reasons for calling the OOH, both for patients with chronic diseases and for the remaining population. The RFEs for patients with heart disease, lung disease, and psychiatric disease tended to relate to symptoms caused by the underlying the diseases. The RFEs for patients with diabetes were similar to those of the remaining population, while the RFEs for patients with cancer were mostly due to lung symptoms, abdominal pains, or death for all four types of contact.

**Table 5:** **The five most common reasons for encounter according to ICPC-2 classification for each contact type**^**1**^

			**Reasons for encounter (n (%))**									
	**Telephone consultation**^**2**^			**Telephone referral**^**3**^			**Clinical consultation**^**4**^			**Home visit**^**5**^		
	**(n = 3,133)**			**(n = 2,177)**			**(n = 4,052)**			**(n = 5,867)**		
**Heart**	Non-specific RFEs, general (A69)	9	(11.7)	Shortness of breath/dyspnoea (R02)	14	(19.7)	Chest pain (A11)	5	(9.8)	Shortness of breath/dyspnoea (R02)	48	(12.8)
	Chest pain (A11)	7	(9.1)	Chest pain (A11)	4	(5.6)	Knee symptom (L15)	4	(7.8)	Chest pain (A11)	31	(6.8)
	Medication prescription, general (A50)	3	(4.1)	Leg symptom (L14)	4	(5.6)	Shortness of breath/dyspnoea (R02)	3	(5.9)	Fever (A03)	24	(6.4)
	Arm symptom (L09)	3	(4.1)	Breathing problem (R04)	3	(5.6)	Cough (R05)	3	(5.9)	Abdominal pain (D01)	19	(4.5)
	Psychological symptoms (P29)	2	(2.1)	Cough (R05)	3	(5.6)	Laceration (S18)	3	(5.9)	Non-specific RFEs, general (A69)	14	(3.5)
**Lung**	Medication prescription, lung (R50)	17	(5.5)	Shortness of breath/dyspnoea (R02)	51	(18.1)	Cough (R05)	28	(8.9)	Shortness of breath/dyspnoea (R02)	168	(14.7)
	Non-specific RFEs, general (A69)	15	(4.3)	Fever (A03)	25	(8.5)	Shortness of breath/dyspnoea (R02)	20	(6.7)	Fever (A03)	105	(9.9)
	Medication prescription, general (A50)	11	(3.6)	Cough (R05)	19	(6.0)	Fever (A03)	19	(6.0)	Cough (R05)	75	(6.5)
	Shortness of breath/dyspnoea (R02)	11	(3.6)	Breathing problem (R04)	17	(5.7)	Throat symptom (R21)	15	(5. 0)	Breathing problem (R04)	53	(4.5)
	Chest pain (A11)	10	(2.7)	Abdominal pain (D01)	13	(4.3)	Abdominal pain (D01)	14	(3.9)	Death (A96)	49	(4.0)
**Diabetes**	Medication prescription, endocrine (T50)	13	(4.6)	Fever (A03)	22	(8.1)	Fever (A03)	13	(5.4)	Shortness of breath/dyspnoea (R02)	90	(8.6)
	Non-specific RFEs, general (A69)	13	(4.5)	Shortness of breath/dyspnoea (R02)	15	(5.6)	Rash localised (S06)	12	(5.0)	Fever (A03)	60	(5.6)
	Medication prescription, general (A50)	12	(3.6)	Abdominal pain (D01)	14	(5.2)	Throat symptom (R21)	11	(4.6)	Death (A96)	52	(5.3)
	Death (A96)	10	(3.0)	Cough (R05)	9	(3.6)	Cough (R05)	10	(4.2)	Cough (R05)	46	(4.2)
	Leg symptom (L14)	9	(2.6)	Leg symptom (L14)	9	(3.3)	Laceration (S18)	10	(4.2)	Abdominal pain (D01)	42	(3.9)
**Psychiatry**	Psychological symptom (P29)	12	(8.8)	Psych. symptom (P29)	8	(11.0)	Fever (A03)	4	(7.1)	Psychological symptom (P29)	41	(18.3)
	Medication prescription, psychiatry (P50)	12	(8.2)	Fever (A03)	4	(7.0)	Teeth/gum symptom (D19)	4	(7.1)	Feeling anxious (P01)	13	(6.7)
	Non-specific, psychological (P69)	11	(7.5)	Depressed (P03)	3	(5.3)	Throat symptom (R21)	4	(7.1)	Feeling depressed (P03)	10	(4.6)
	Feeling anxious (P01)	9	(7.5)	Abdominal pain (D01)	3	(4.1)	Laceration (S18)	3	(5.5)	Feeling angry (P04)	9	(4.1)
	Non-specific RFEs, general (A69)	8	(6.1)	Chest pain (A11)	2	(4.1)	Abdominal pain localized (D06)	3	(5.5)	Chest pain (A11)	8	(3.4)
**Cancer**	Death (A96)	12	(3.9)	Fever (A03)	23	(8.1)	Throat symptom (R21)	23	(8.5)	Death (A96)	107	(11.2)
	Non-specific RFEs, general (A69)	10	(3.5)	Shortness of breath/dyspnoea (R02)	21	(7.4)	Fever (A03)	14	(5.2)	Fever (A03)	82	(8.2)
	Medication prescription, general (A50)	9	(3.2)	Abdominal pain (D01)	19	(6.7)	Cough (R05)	13	(4.8)	Abdominal pain (D01)	60	(5.8)
	Abdominal pain (D01)	8	(2.8)	Death (A96)	17	(6.3)	Abdominal pain (D01)	11	(4.4)	Shortness of breath/dyspnoea (R02)	58	(5.1)
	Fever (A03)	7	(2.1)	Cough (R05)	9	(3.5)	Abdominal pain localized(D06)	11	(4.3)	Cough (R05)	41	(4.1)
**Others**	Abdominal pain (D01)	76	(3.5)	Fever (A03)	103	(7.4)	Throat symptom (R21)	260	(8.5)	Fever (A03)	213	(7.9)
	Fever (A03)	65	(3.0)	Abdominal pain (D01)	91	(6.2)	Fever (A03)	171	(5.6)	Abdominal pain (D01)	193	(6.4)
	Medication prescription, general (A50)	58	(2.7)	Throat symptom (R21)	77	(5.6)	Cough (R05)	143	(4.7)	Death(A96)	93	(3.7)
	Headache (N01)	57	(2.7)	Laceration (S18)	48	(3.6)	Abdominal pain (D01)	121	(4.0)	Cough (R05)	86	(3.2)
	Non-specific RFEs, general (A69)	56	(2.6)	Cough (R05)	43	(3.0)	Laceration (S18)	97	(3.7)	Abdominal pain epigastric (D02)	85	(3.1)

^*1*^*N*umber of identified patients for each contact type is calculated without consideration of patients with more than one of the chronic disease. ^2^ A total of 2,953 unique patients. Referrals to hospitals or other institutions are included in telephone consultations. ^4^A total of 3,853 unique patients. ^5^A total of 5,091 unique patients.

The five most frequent diagnoses stated by the GPs reflected characteristic symptoms of the chronic diseases, except for diabetes (Table 6) for which the recorded diagnoses were comparable with variations in diagnoses found in the remaining population. The diagnoses stated for cancer patients were characterized by infection, pain, and death.

**Table 6:** **The five most common diagnoses according to the ICPC-2 classification for each contact type**^**1**^

		**Diagnoses (n (%))**										
	**Telephone consultation**^**2**^			**Telephone referral**^**3**^			**Clinical consultation**^**4**^			**Home visit**^**5**^		
	**(n = 3,133)**			**(n = 2,177)**			**(n = 4,052)**			**(n = 5,867)**		
**Heart**	Acute myocardial infarction (K75)	9	(16.7)	Chronic obstructive pulm. dis. (R95)	7	(12.7)	Chronic obstructive pulm. dis. (R95)	6	(11.3)	Chronic obstructive pulm. dis. (R95)	40	(9.5)
	Cystitis (U71)	7	(9.9)	Pneumonia (R81)	6	(10.9)	Acute myocardial infarction (K75)	3	(5.6)	Pneumonia (R81)	33	(8.1)
	Endocrine/nutrit. disorder (T99)	5	(7.1)	Angina (K74)	4	(6.9)	Laceration (S18)	3	(5.6)	Heart failure (K77)	25	(6.4)
	Pain general (A01)	4	(5.3)	Effect pros. device (A89)	4	(6.9)	Disease digestive syst. (D99)	2	(3.7)	Acute myocardial infarction (K75)	19	(5.0)
	Medication prescription, general (A50)	3	(4.7)	Heart failure (K77)	3	(5.2)	Conjunctivitis infect. (F70)	2	(3.7)	Ischaemic heart disease (K74)	18	(3.7)
**Lung**	Asthma (R96)	48	(15.3)	Chronic obstructive pulm. dis. (R95)	44	(16.4)	Pneumonia (R81)	30	(8.9)	Chronic obstructive pulm. dis. (R95)	282	(23.3)
	Chronic obstructive pulm. dis. (R95)	43	(14.2)	Pneumonia (R81)	37	(13.6)	Chronic obstructive pulm. dis. (R95)	26	(7.2)	Pneumonia (R81)	156	(12.5)
	Medication prescription, general (A50)	20	(6.0)	Asthma (R96)	19	(7.0)	Asthma (R96)	24	(6.9)	Death (A96)	40	(4.1)
	Gastroent. Infect. (D73)	7	(2.5)	Effect pros. device (A89)	6	(2.1)	Cystitis (U71)	13	(3.6)	Asthma (R96)	27	(3.2)
	Alcohol abuse(P15)	6	(2.1)	Shortness of breath/dyspnoea (R02)	5	(1.9)	Abdominal pain (D01)	10	(2.9)	Heart failure (K77)	23	(2.3)
**Diabetes**	Endocrine/nutrit. disorder (T99)	26	(9.8)	Pneumonia (R81)	24	(9.6)	Pneumonia (R81)	15	(5.6)	Pneumonia (R81)	103	(9.2)
	Cystitis (U71)	12	(3.5)	Chronic obstructive pulm. dis. (R95)	10	(4.2)	Skin infect. (S76)	10	(4.1)	Chronic obstructive pulm. dis. (R95)	86	(7.1)
	Medication prescription, general (A50)	10	(3.0)	Infectious disease (A78)	9	(3.9)	Laceration (S18)	8	(3.2)	Death (A96)	53	(5.1)
	Death (A96)	9	(3.0)	General dis.(A99)	9	(3.9)	Upper resp. Infect. (R74)	7	(2.8)	Heart failure (K77)	40	(3.5)
	Adverse effect medical agent (A85)	9	(3.0)	Digestive symptoms (D99)	8	(3.7)	Gastroent. Infect. (D73)	6	(2.5)	Cystitis (U71)	31	(2.7)
**Psychiatry**	Schizophrenia (P72)	7	(9.1)	Schizophrenia (P72)	6	(10.5)	Teeth/gum symptom (D19)	3	(5.1)	Schizophrenia (P72)	24	(10.4)
	Psychosis (P98)	5	(8.2)	Pneumonia (R81)	4	(7.2)	Schizophrenia (P72)	3	(5.1)	Affective psychosis (P73)	13	(5.6)
	Psychological disorders (P99)	4	(5.4)	Skin infect. (S11)	3	(5.5)	Tonsillitis ac. (R76)	2	(4.0)	Chronic obstructive pulm. dis. (R95)	13	(5.6)
	Chronic alcohol abuse (P15)	3	(4.1)	Psychological sympt. (P29)	2	(4.1)	Laceration (S18)	2	(4.0)	Psychosis (P98)	12	(5.4)
	Anxiety disorder (P74)	3	(4.1)	Psychosis (P98)	2	(4.1)	Cystitis (U71)	2	(4.0)	Anxiety disorder (P74)	10	(4.3)
**Cancer**	Cystitis (U71)	25	(8.9)	Pneumonia (R81)	24	(9.4)	Pneumonia (R81)	13	(4.7)	Pneumonia (R81)	115	(10.5)
	Death (A96)	18	(6.5)	Death (A96)	17	(6.6)	Tonsilitis acute (R76)	11	(4.3)	Death (A96)	106	(10.1)
	Medication prescription, general (A50)	10	(2.9)	Abdominal pain (D01)	9	(3.5)	Cystitis (U71)	10	(3.9)	Chronic obstructive pulm. dis. (R95)	45	(4.1)
	Abdominal pain (D01)	10	(2.9)	Chronic obstructive pulm. dis. (R95)	8	(3.2)	Laceration (S18)	10	(3.9)	Disease digestive system (D99)	42	(3.8)
	General disease (A99)	10	(2.9)	Urinary retention (U08)	8	(3.2)	Skin infection post traumatic (S11)	8	(3.6)	General disease (A99)	37	(3.1)
**Others**	Cystitis (U71)	88	(6.3)	Pneumonia (R81)	82	(6.4)	Pneumonia (R81)	121	(3.9)	Pneumonia (R81)	212	(7.6)
	Gastroenteritis pres. Infect. (D73)	82	(5.9)	Tonsillitis acuta (R76)	81	(6.3)	Tonsillitis acute (R76)	118	(3.8)	Gastroenteritis pres. Infect. (D73)	114	(4.3)
	Conjunctivitis Infectious (F70)	46	(3.3)	Abdominal pain (D01)	43	(3.3)	Upper resp. Infect. Acute(R74)	109	(3.4)	Migraine (N89)	91	(3.2)
	Medication prescription, general (A50)	43	(3.1)	Laceration (S18)	42	(3.2)	Laceration (S18)	104	(3.3)	Cholecystitis (D98)	91	(3.0)
	Influenza (R80)	42	(3.0)	Cystitis (U71)	42	(3.2)	Strep throat (R72)	103	(3.2)	Death (A96)	88	(2.8)

^1^Number of identified patients for each contact type is calculated without consideration of patients with more than one of the chronic diseases. ^2^A total of 2,953 unique patients. ^3^A total of 2,033 unique patients. Referral to hospitals or other institutions are included in telephone consultations. ^4^A total of 3,853 unique patients. ^5^A total of 5,091 unique patients.

## Discussion

One third of the patients contacting the OOH had a history of at least one of the selected chronic diseases. The RFE was related to the underlying chronic condition for one in four of the patients with heart disease, lung disease, diabetes, and cancer, and one in three of the patients with a psychiatric disease. Patients with a chronic disease were more likely to be referred to a face-to-face encounter than the remaining population, except for patients diagnosed with a psychiatric disease who were more often completed by telephone. Among the patients with chronic diseases, patients with heart disease were most often referred to hospital from the OOH. Both the RFE and the diagnosis assigned by the GP were generally related to the patient’s chronic diseases, except for patients with diabetes for whom symptoms and diagnosis were similar to the remaining patient population.

### Strengths and weaknesses

The study design and the sampling method secured data from a large number of randomly selected and consecutive patients who were representative of the entire population of patients contacting the OOH services. In total, 95.5% of all 8-hour duty periods handled by the OOH telephone centre were included in the study. This extensive data set also provides a relatively high overall statistical precision. However, the statistical precision is affected when data are stratified into smaller groups. Therefore, a larger population will be required in future research to increase the statistical precision in smaller groups.

GPs had to answer a questionnaire immediately after completion of each individual contact, and this time minimisation is expected to reduce recall bias. The data were generated electronically to ensure accuracy in data transmission and correlation with registers used for identification of the study population. The Danish CPR numbers allowed us to link each patient with national Danish registries [24-26].

For identifying patients according to the broader term ‘chronic lung disease’, we used a validated algorithm developed to identify patients aged 35 years or older with Chronic Obstructive Pulmonary Disease (COPD) [23]. The algorithm presents a positive predictive value (PPV) of 30-97%, varying with age; the lowest PPV value is explained by the inclusion of many younger patients with asthma. Because of the age span in our population, we expect to meet the terms for inclusion of the youngest patients with asthma. The high PPVs shown for ICD-10 diagnoses of heart disease, diabetes, and cancer (98-100%) support our method for identification of patients [27]. The method used for identification of patients with psychiatric diseases is in line with the acknowledged tradition of using selected diagnostic codes for research [28].

The gender distributions for diagnoses included in the study correspond to the national figures [29]; males account for the majority of heart diseases and diabetes, while women account for the majority of psychiatric diseases. In contrast to official statistics, females in our study population accounted for the majority of contacts regarding lung diseases and cancer, which may result from females’ more frequent use of the health care system in general, including the OOH [11,30].

### Comparison with other studies

Den Boer et al. found a strong association between chronic disease and use of OOH, and patients with exacerbation of a chronic disease were also found to form an intrinsic part of the demand for OOH services [31]. In our study, between one in four and one in three of all contacts from patients with chronic diseases were related to en exacerbation of a pre-existing condition compared to one in ten among the rest of the patients. These figures indicate that the chronic diseases in our study required greater attention and increased follow-up in the OOH setting. However, our study cannot reveal whether some of the contacts due to exacerbation could be prevented. If an exacerbation of a chronic disease is considered potentially preventable through regular daytime care, these contacts to the OOH services could indicate challenging issues in daytime care. Yet, the many contacts could also indicate that patients with chronic diseases are heavy users of the health care system at any time of the day, as also concluded in previous studies [1,10].

Patients with chronic diseases were more likely to be referred to hospital or ED than the remaining population, except for patients with an exacerbation of diabetes. Diabetes is increasingly followed-up in general practice on the basis of guidelines, and the results of this study may suggest a positive implication of this development [32,33]. Dusheiko et al found that an intensive effort to upskill patients from ‘poor’ to ‘moderate’ diabetic control is linked with a reduction in hospital admissions [34].

Our study showed a high number of referrals to hospital for patients with heart disease who called the OOH due to a new health problem. Noman et al. found that patients having an acute myocardial infarction outside daytime experienced a significantly lower history of angina pectoris prior to an event [35]. Therefore, such a patient history could be interpreted as a new event rather than an exacerbation of an existing event. This is a likely explanation for our results as myocardial infarction, in our study, is one of the most frequent diagnoses resulting from a call to the OOH.

Our study showed that the most frequent RFEs for each of the diagnostic groups also reflected symptoms that are characteristic for the five selected chronic diseases. This connection was supported by the diagnoses suggested by the GP at the end of the contact, which increases the statistical precision of the analyses. In an unspecified sample of 295 contacts to the OOH services in Switzerland, Huber et al found a broad variation of unspecific symptoms in the RFEs, which calls for more meticulous research in a larger population sample [13]. Johansen et al found that patients contacting the OOH services due to mental illness were generally managed in the OOH casualty clinics without referral to psychiatric inpatient care [36]. This might reflect our finding that 57.3% of all contacts regarding psychiatric symptoms were handled by telephone consultation alone.

## Conclusions

We found that one third of a population randomly sampled by their call to the OOH had one or more of the five chronic diseases selected for the study. The RFEs and the diagnoses for these groups of patients were largely related to the already diagnosed chronic diseases. Compared to the remaining population, patients in these five diagnostic groups were more often than other patients managed by the GPs in the OOH services. Our new findings regarding five highly incident chronic diseases contribute with important knowledge to the field, which should be further researched in future studies on chronic care.

Across the world, populations are getting older. This development puts increased pressure on the health care resources. Sufficient chronic care must be coordinated all day round [37], and the results of the present study outline focal points for future daytime care for these patients. However, more research is required to explore the correlation between daytime care and the demand for the OOH services among patients who experience exacerbation of a chronic disease.

## Competing interest

The authors declare that they have no competing interest.

## Authors’ contributions

LF contributed to research design, data collection, analyses, and led the draft of the manuscript. GM contributed to data collection and drafting of the manuscript. MBC and FO contributed to research design and drafting of the manuscript. MV contributed to the data collection and to the research design, and drafting of the manuscript. PV contributed on the research design and drafting of the manuscript. All authors read and approved the final manuscript.

## Pre-publication history

The pre-publication history for this paper can be accessed here:

http://www.biomedcentral.com/1471-2296/15/114/prepub

## References

[B1] Danish Health and Medicines Authority Jørgensen SJ Kronisk sygdom. Patient, sundhedsvæsen og samfund Chronic disease. Patient, healthcare system and society 2005 Copenhagen: Danish Health and Medicines Authority

[B2] Danish Health and Medicines Authority Forløbsprogrammer for kronisk sygdom – Generisk model og Forløbsprogram for diabetes. 1.0 ed Chronic disease self-management programmes - the generic model on diabetes 2008 Copenhagen: Danish Health and Medicines Authority

[B3] Danish Health and Medicines Authority KOL - Kronisk Obstruktiv Lungesygdom, Anbefalinger for tidlig opsporing, opfølgning, behandling og rehabilitering COPD - Chronic Obstructive Lung Disease. Recommendations on early detection, follow-up and rehabilitation 2007 20 Copenhagen: Danish Health and Medicines Authority

[B4] Danish Health and Medicines Authority Referenceprogram for unipolar depression hos voksne: udarbejdet af en arbejdsgruppe nedsat af Sekretariatet for Referenceprogrammer - SfR Reference programme on depression among adults, produced by a working group at the Danish Health and Medicines Authority 2007 Copenhagen: Danish Health and Medicines Authority

[B5] Danish Health and Medicines Authority Referenceprogram for angstlidelser hos voksne: udarbejdet af en arbejdsgruppe nedsat af Sekretariatet for Referenceprogrammer - SfR Reference programme on anxiety among adults, produced by the Danish Health and Medicines Authority 2007 Copenhagen: Danish Health and Medicines Authority

[B6] Barnett K Mercer SW Norbury M Watt G Wyke S Guthrie B Epidemiology of multimorbidity and implications for health care, research, and medical education: a cross-sectional study Lancet 2012 380 9836 37 43 10.1016/S0140-6736(12)60240-2 22579043

[B7] Frank Hansen M Haagen Pedersen L Sundhedsudgifter og finanspolitisk holdbarhed. [Health expenses and fiscal durability] Dan Econ Soc J 2010 148 1 21 42

[B8] Christensen MB Olesen F Out of hours service in Denmark: evaluation five years after reform BMJ 1998 316 7143 1502 1505 10.1136/bmj.316.7143.1502 9582141 PMC28553

[B9] Olesen F Jolleys JV Out of hours service: the Danish solution examined BMJ 1994 309 1624 1626 10.1136/bmj.309.6969.1624 7819945 PMC2542018

[B10] World Health Organization Innovative care for chronic conditions. S.l.: Noncommunicable Diseases and Mental Health 2002 Geneva: World Health Organization

[B11] Christensen MB Frequent attenders at the out of hours service in Denmark - Implementation of change in general practice A stratified cluster randomised controlled trial among frequent attenders at the out of hours service in the County of Northern Jutland, Denmark 2001 Aarhus: PhD thesis. Aarhus University, Faculty of Health Sciences

[B12] Moth G Olesen F Vedsted P Reasons for encounter and disease patterns in Danish primary care: changes over 16 years Scand J Prim Health Care 2012 30 2 70 75 10.3109/02813432.2012.679230 22643150 PMC3378007

[B13] Huber CA Rosemann T Zoller M Eichler K Senn O Out-of-hours demand in primary care: frequency, mode of contact and reasons for encounter in Switzerland J Eval Clin Pract 2011 17 1 174 179 10.1111/j.1365-2753.2010.01418.x 20831666

[B14] Statistic Denmark http://www.statistikbanken.dk/statbank5a/default.asp?w?=?1920

[B15] Olivarius NF Hollnagel H Krasnik A Pedersen PA Thorsen H The Danish National Health Service Register. A tool for primary health care research Dan Med Bull 1997 44 4 9377908

[B16] Flarup L Moth G Christensen MB Vestergaard M Olesen F Vedsted P A feasible method to study the Danish out-of-hours primary care service Dan Med J 2014 61 5 A4847 24814746

[B17] Bentsen BG International classification of primary care Scand J Prim Health Care 1986 4 1 43 50 10.3109/02813438609013970 3961309

[B18] Lynge E Sandegaard JL Rebolj M The Danish national patient register Scand J Publ Health 2011 39 7 Suppl 30 33 10.1177/1403494811401482 21775347

[B19] Carstensen B Kristensen JK Marcussen MM Borch-Johnsen K The national diabetes register Scand J Publ Health 2011 39 7 suppl 58 61 10.1177/1403494811404278 21775353

[B20] Mors O Perto GP Mortensen PB The Danish psychiatric central research register Scand J Publ Health 2011 39 7 suppl 54 57 10.1177/1403494810395825 21775352

[B21] Storm HH Michelsen EV Clemmensen IH Pihl J The Danish cancer registry-history, content, quality and use Dan Med Bull 1997 44 535 539 9408738

[B22] Gjerstorff ML The Danish cancer registry Scand J Publ Health 2011 39 7 suppl 42 45 10.1177/1403494810393562 21775350

[B23] Smidth M Sokolowski I Kaersvang L Vedsted P Developing an algorithm to identify people with Chronic Obstructive Pulmonary Disease (COPD) using administrative data BMC Med Inform Decis Mak 2012 12 38 10.1186/1472-6947-12-38 22616576 PMC3444358

[B24] Pedersen CB Gøtzsche H Møller JØ Mortensen PB The Danish civil registration system Dan Med Bull 2006 53 441 449 17150149

[B25] Mosbech J Jorgensen J Madsen M Rostgaard K Thornberg K Poulsen TD The national patient registry. Evaluation of data quality Ugeskr Laeger 1995 157 26 3741 3745 7631448

[B26] Thygesen LC Ersboll AK Danish population-based registers for public health and health-related welfare research: introduction to the supplement Scand J Publ Health 2011 39 7 Suppl 8 10 10.1177/1403494811409654 21775344

[B27] Thygesen SK Christiansen CF Christensen S Lash TL Sorensen HT The predictive value of ICD-10 diagnostic coding used to assess Charlson comorbidity index conditions in the population-based Danish National Registry of Patients BMC Med Res Methodol 2011 11 83 10.1186/1471-2288-11-83 21619668 PMC3125388

[B28] International Advisory Group for the Revision of ICD-10 Mental and Behavioural Disorders A conceptual framework for the revision of the ICD-10 classification of mental and behavioural disorders World Psychiatr 2011 10 86 92 10.1002/j.2051-5545.2011.tb00022.x PMC3104876 21633677

[B29] Denmark S Mænd & kvinder 2011. [Men & women 2011] 2011 Statistics Denmark: Copenhagen

[B30] Folkesundhedsrapporten Danmark 2007 Report on Public Health in Denmark 2007 2007 Copenhagen: National Institute of Public Health

[B31] den Boer-Wolters D Knol MJ Smulders K de Wit NJ Frequent attendance of primary care out-of-hours services in the Netherlands: characteristics of patients and presented morbidity Fam Pract 2010 27 2 129 134 10.1093/fampra/cmp103 20032165

[B32] Hansen LJ Siersma V Beck-Nielsen H de Fine ON Structured personal care of type 2 diabetes: a 19 year follow-up of the study Diabetes Care in General Practice (DCGP) Diabetologia 2013 56 6 1243 1253 10.1007/s00125-013-2893-1 23549519

[B33] Schroll H Christensen RD Thomsen JL Andersen M Friborg S Sondergaard J The danish model for improvement of diabetes care in general practice: impact of automated collection and feedback of patient data Int J Fam Med 2012 Article ID 208123: 5 10.1155/2012/208123 PMC3409523 22888424

[B34] Dusheiko M Doran T Gravelle H Fullwood C Roland M Does higher quality of diabetes management in family practice reduce unplanned hospital admissions? Health Serv Res 2011 46 1 Pt 1 27 46 20880046 10.1111/j.1475-6773.2010.01184.x PMC3034260

[B35] Noman A Ahmed JM Spyridopoulos I Bagnall A Egred M Mortality outcome of out-of-hours primary percutaneous coronary intervention in the current era Eur Heart J 2012 33 24 3046 3053 10.1093/eurheartj/ehs261 22947609

[B36] Johansen IH Morken T Hunskaar S How Norwegian casualty clinics handle contacts related to mental illness: a prospective observational study Int J Ment Health Syst 2012 6 1 3 10.1186/1752-4458-6-3 22520067 PMC3434113

[B37] Coleman K Austin BT Brach C Wagner EH Evidence on the chronic care model in the new millennium Health Aff (Millwood) 2009 28 1 75 85 10.1377/hlthaff.28.1.75 19124857 PMC5091929

